# Acid-sensing ion channel 1a contributes to hippocampal LTP inducibility through multiple mechanisms

**DOI:** 10.1038/srep23350

**Published:** 2016-03-21

**Authors:** Ming-Gang Liu, Hu-Song Li, Wei-Guang Li, Yan-Jiao Wu, Shi-Ning Deng, Chen Huang, Oleksandr Maximyuk, Volodymyr Sukach, Oleg Krishtal, Michael X. Zhu, Tian-Le Xu

**Affiliations:** 1 Discipline of Neuroscience and Department of Anatomy, Histology and Embryology, Collaborative Innovation Center for Brain Science, Shanghai Jiao Tong University School of Medicine, Shanghai 200025, China; 2 Department of Developmental and Behavioral Pediatrics, Shanghai Children’s Medical Center, Shanghai Jiao Tong University School of Medicine, Shanghai 200129, China; 3 Bogomoletz Institute of Physiology of NAS Ukraine, 4 Bogomoletz Str., 01024 Kyiv, Ukraine; 4 State Key Laboratory for Molecular and Cellular Biology, 4 Bogomoletz Str., 01024 Kyiv, Ukraine; 5 Department of Integrative Biology and Pharmacology, The University of Texas Health Science Center at Houston, 6431 Fannin St., Houston, TX 77030, USA

## Abstract

The exact roles of acid-sensing ion channels (ASICs) in synaptic plasticity remain elusive. Here, we address the contribution of ASIC1a to five forms of synaptic plasticity in the mouse hippocampus using an *in vitro* multi-electrode array recording system. We found that genetic deletion or pharmacological blockade of ASIC1a greatly reduced, but did not fully abolish, the probability of long-term potentiation (LTP) induction by either single or repeated high frequency stimulation or theta burst stimulation in the CA1 region. However, these treatments did not affect hippocampal long-term depression induced by low frequency electrical stimulation or (RS)-3,5-dihydroxyphenylglycine. We also show that ASIC1a exerts its action in hippocampal LTP through multiple mechanisms that include but are not limited to augmentation of NMDA receptor function. Taken together, these results reveal new insights into the role of ASIC1a in hippocampal synaptic plasticity and the underlying mechanisms. This unbiased study also demonstrates a novel and objective way to assay synaptic plasticity mechanisms in the brain.

Acid-sensing ion channels (ASICs) are proton-gated members of the degenerin/epithelial sodium channel family^1^,^2^. To date, at least six ASIC subunits have been identified: 1a, 1b, 2a, 2b, 3, and 4^3^. ASIC1a is widely distributed in many brain regions with high synaptic density and is a key sensor for extracellular acidosis in the central nervous system^4^,^5^. It is well documented that ASIC1a critically contributes to a variety of physiological and pathological conditions, such as learning and memory^6^, fear^7^,^8^, anxiety^9^,^10^, pain perception^11^,^12^, and ischemic neuronal injury^13^,^14^. However, the synaptic basis for the involvement of ASIC1a in these processes remains elusive.

Synaptic plasticity, including long-term potentiation (LTP) and long-term depression (LTD), is critical for a broad range of brain behaviors and neurological disorders^15^,^16^,^17^. Structurally, ASIC1a is enriched in the postsynaptic membrane and shown to affect the density of dendritic spines^18^. Functionally, ASIC1a has also been demonstrated to be important for LTP induction in the amygdala, which is required for fear learning and memory^19^. Therefore, it has been interestingly proposed that ASIC1a is critically involved in synaptic plasticity at some central synapses, which might set the foundation for their physiological and pathological functions. However, the exact role of ASIC1a in synaptic transmission and plasticity remains unclear and controversial^20^.

In the present study, we evaluated the role of ASIC1a in LTP and LTD induction in the hippocampus by using a 64-channel multi-electrode dish (MED64) recording system^21^. The introduction of the multi-electrode array recording technique brings in a new and broad dimension into the synaptic plasticity field, allowing one to address the probabilistic nature of LTP or LTD inducibility at multiple sites within a local network both simultaneously and objectively^22^,^23^. We found that genetic deletion or pharmacological blockade of ASIC1a reduced, but not fully abolished, the induction of hippocampal LTP in a protocol-independent manner, while leaving LTD unaffected. Although virus-mediated expression of ASIC1a in the hippocampus fully rescued the impaired LTP in the ASIC1a null mice, bath application of D-cycloserine (DCS), a co-agonist of the NMDA receptor (NMDAR) at the glycine binding site^24^, or low Mg^2+^ treatment only partially restored the ability of hippocampal synapses to undergo LTP. Taken together, these results provide a novel and objective assessment of the role of ASIC1a in hippocampal LTP/LTD and suggest that multiple mechanisms might underlie the involvement of ASIC1a in synaptic plasticity in the brain.

## Results

### Genetic deletion of ASIC1a reduces the probability of LTP induction in the hippocampus

**Figure 1:**
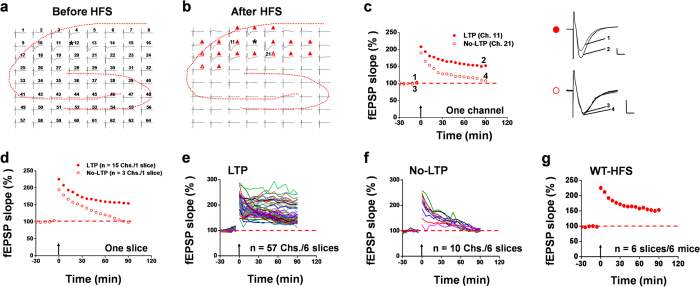
Multi-channel recordings of HFS-evoked LTP in hippocampal slices from WT mice. (**a,b**) Overview of multisite synaptic responses recorded at baseline (**a**) and 90 min after HFS (**b**) in a representative hippocampal slice taken from a WT mouse. Asterisks indicate the stimulated channel (Ch. 12). All LTP channels are marked as filled red triangle, while all No-LTP channels marked as open red triangle in (**b**). The activated channels in this slice exhibited more LTP than No-LTP response following HFS. (**c**) Representative time courses of normalized fEPSP slope for the LTP (Ch. 11, (**a)** filled red circle) and No-LTP (Ch. 21, (**b**) open red circle) channels as marked in (**b**). Inset: representative fEPSP traces at the time points indicated by the numbers. Calibration: upper, 100 μV, 10 ms; lower, 50 μV, 10 ms. (**d**) Averaged data for the 15 LTP channels and 3 No-LTP channels in the same slice shown in (**a–c**). (**e,f**) Pooled data for fEPSP slope changes of all 57 LTP channels (**e**) and 10 No-LTP channels (**f**) in 6 slices from 6 WT mice. (**g**) Time course of mean fEPSP slope changes of all activated channels in the 6 slices from 6 WT mice. The mean fEPSP slope during the baseline period was −39.8 μV/ms. Arrows in (**c–g**) indicate the HFS delivery. Error bars in (**g**) (many are smaller than the symbols) represent SEM.

Although ASIC1a is widely expressed in the mammalian brain, it remains controversial whether it plays an important role in long-lasting synaptic plasticity in those regions with high synaptic density^20^. Using the multi-channel recording system, we sought to evaluate the role of ASIC1a in hippocampal LTP inducibility. Before doing that, we first checked whether ASIC1a has any effect on basal synaptic transmission in the hippocampus. Analysis of the input/output relationship, in terms of either field excitatory postsynaptic potentials (fEPSP) slope or the number of activated channels, failed to detect any difference between wild-type (WT) and ASIC1a knockout (KO) mice (Supplementary Fig. S1), suggesting no role of ASIC1a in basal synaptic transmission. Paired-pulse facilitation, a form of short-term plasticity^25^, was also indistinguishable between WT and ASIC1a KO neurons at any interval tested (Supplementary Fig. S1), arguing against any effect of ASIC1a deletion on the probability of presynaptic neurotransmitter release. We then studied the LTP induction properties by delivering a high frequency stimulation (HFS, 100 Hz, 1 s) protocol to CA1 synapses. As shown in Fig. 1a,b, HFS elicited clear LTP in most of the activated channels, which lasted for at least 90 min (marked by filled red triangle in Fig. 1b and exemplified for Ch. 11 in Fig. 1c). These are referred to as LTP channels. However, in response to the same HFS protocol, a small number of channels developed immediate potentiation but then recovered to the baseline over time (marked by open red triangle in Fig. 1b and exemplified for Ch. 21 in Fig. 1c). These are referred to as No-LTP channels. For the slice shown in Fig. 1a–c, the 15 LTP channels had a mean fEPSP slope at 90 min after HFS equivalent to 154.1% of their baseline values and the 3 No-LTP channels exhibited a mean fEPSP slope at 90 min after HFS similar to their baseline value (98.3%, Fig. 1d). Pooling all data together from a total of 6 slices from 6 mice, we found 57 LTP channels (Fig. 1e), but only 10 No-LTP channels (Fig. 1f). Calculating the mean values for individual slices, we obtained, on average, 11.7 ± 1.7 LTP channels out of 14.0 ± 1.3 total activated channels per slice and an induction ratio of 82.5 ± 7.4% (n = 6 slices/6 mice). For all channels included in Fig. 1e,f, the potentiation of synaptic responses was 153.0 ± 4.5% of baseline at 90 min after HFS delivery to the CA1 region of the WT mice (Fig. 1g, n = 6 slices/6 mice, *P* < 0.001 vs. baseline, paired t-test).

**Figure 2:**
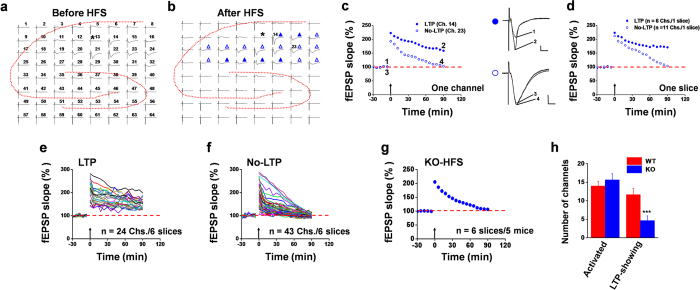
Genetic deletion of ASIC1a reduces the probability of LTP induction by HFS in the hippocampus. (**a,b**) Overview of multisite synaptic responses recorded at baseline (**a**) and 90 min after HFS (**b**) in a representative hippocampal slice taken from an ASIC1a KO mouse. Asterisks indicate the stimulated channel (Ch. 13). All LTP channels are marked as filled blue triangle, while all No-LTP channels marked as open blue triangle in (**b**). The activated channels in this slice exhibited more No-LTP than LTP response following HFS. (**c**) Representative time courses of normalized fEPSP slope for the LTP (Ch. 14, **a**, filled blue circle) and No-LTP (Ch. 23, **b**, open blue circle) channels as marked in (**b**). Inset: representative fEPSP traces at the time points indicated by the numbers. Calibration: 100 μV, 10 ms. (**d**) Averaged data from the 6 LTP channels and 11 No-LTP channels in the same slice shown in (**a–c**). (**e,f**) Pooled data for fEPSP slope changes of all 24 LTP channels (**e**) and 43 No-LTP channels (**f**) in 6 slices from 5 ASIC1a KO mice. Note, for display purposes, some No-LTP channels are not shown in (**f**) due to their extraordinarily high magnitude of acute potentiation or large fluctuation. (**g**) Time course of mean fEPSP slope changes of all activated channels in the 6 slices from 5 ASIC1a KO mice. The mean fEPSP slope during the baseline period was −40.7 μV/ms. (**h**) Summary data (number/slice, n = 6 slices/5–6 mice) of activated channels that responded to Schaffer collateral pathway stimulation and LTP channels that responded to the HFS protocol in CA1 regions of WT (red) and ASIC1a KO (blue) mice. ***P < 0.001 for ASIC1a KO vs. WT, unpaired t-test. Arrows in c-g indicate the HFS delivery. Error bars in (**g**) (many are smaller than the symbols) and (**h**) represent SEM.

Applying the same conditions and criteria, we next examined hippocampal slices from ASIC1a KO mice. Although some LTP channels (marked by filled blue triangle in Fig. 2b and exemplified for Ch. 14 in Fig. 2c) were also obtained, most of the activated channels displayed the No-LTP response (marked by open blue triangle in Fig. 2b and exemplified for Ch. 23 in Fig. 2c). For the slice shown in Fig. 2a–c, the 6 LTP channels had a mean fEPSP slope at 90 min after HFS equivalent to 170.9% of their baseline values and the 11 No-LTP channels exhibited a mean fEPSP slope similar to their baseline value (103.6%, Fig. 2d). Pooling all data together from 6 slices, we found just 24 LTP channels (Fig. 2e), but 43 No-LTP channels (Fig. 2f). Since the No-LTP channels dominated in the ASIC1a KO hippocampal slices, the pooled data of all channels revealed a blockade of LTP induction (Fig. 2g, 106.4 ± 2.8% of baseline at 90 min after HFS, n = 6 slices/5 mice, *P* = 0.438 vs. baseline, Wilcoxon Signed Rank test). On average, the number of LTP channels was 4.7 ± 1.3 out of 15.7 ± 1.6 activated channels per slice, mounting to an induction ratio of 29.8 ± 7.7% (n = 6 slices/5 mice) for ASIC1a KO slices, which is significantly lower than that for the WT slices (Fig. 2h, *P* < 0.001, unpaired t-test). These data demonstrate that genetic ablation of ASIC1a greatly reduced the probability of LTP induction in hippocampal neurons.

ASIC1a deletion did not completely abolish HFS-evoked LTP. This raises a question if ASIC1a is absolutely required for the LTP induction or these channels just affect the threshold of LTP induction. To distinguish these possibilities, we applied a stronger LTP protocol (4 × HFS, 100 Hz, 1 s, 4 times at 20 s interval) to the Schaffer collateral pathway in the hope of overcoming the additional barrier of LTP induction due to the loss of ASIC1a. Indeed, the delivery of 4 × HFS increased the probability of LTP induction in both WT and ASIC1a KO mice (compare Supplementary Fig. S2 with Figs 1 and 2). However, the difference in the induction ratio of LTP between WT and ASIC1a KO mice remained to be significant (WT vs. ASIC1a KO: 86.2 ± 3.5% vs. 53.3 ± 8.4%, *P* < 0.01, unpaired t-test, Supplementary Fig. S2). The pooled data of all activated channels revealed a significant reduction in the magnitude of LTP by ASIC1a deletion (WT vs. ASIC1a KO: 171.0 ± 7.3% vs. 127.6 ± 5.3% of baseline at 90 min after 4 × HFS, *P* < 0.001, unpaired t-test, Supplementary Fig. S2). Thus, it does appear that ASIC1a can lower the threshold of LTP induction in at least a certain population of hippocampal neurons, although it cannot be completely ruled out that ASIC1a might also be indispensable for LTP in another population of cells.

Multiple protocols are available to induce LTP in the hippocampal CA1 region, and they may bear different mechanisms in either induction or maintenance phase^26^,^27^. To examine whether the observed deficiency in LTP inducibility in the ASIC1a KO mice was unique to the LTP induction protocol used, we delivered a theta burst stimulation (TBS) protocol (10 bursts of 4 pulses at 100 Hz, with inter-burst intervals of 200 ms) to the CA3-CA1 synapse. We obtained similar results as HFS, whereby ASIC1a deletion rendered the hippocampal neurons less likely to undergo TBS-induced LTP (WT vs. ASIC1a KO: induction ratio, 85.9 ± 6.9% vs. 24.4 ± 8.1%; LTP magnitude, 162.5 ± 4.2% vs. 104.5 ± 3.7% of baseline at 120 min after TBS; Supplementary Fig. S3). Thus, the contribution of ASIC1a to hippocampal LTP inducibility is protocol-independent.

### ASIC1a does not play a critical role in hippocampal LTD

**Figure 3:**
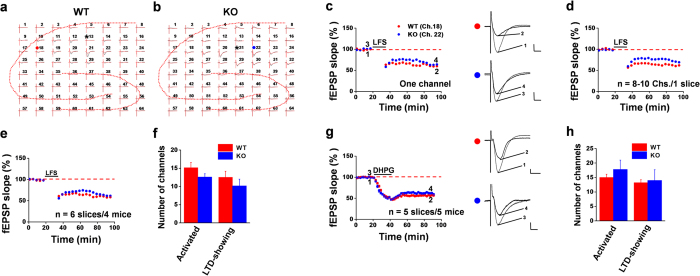
Genetic deletion of ASIC1a does not alter the probability of LTD induction by LFS or DHPG in the hippocampus. (**a,b**) Overview of multisite synaptic responses recorded at baseline (black) and 60 min after LFS (red) in representative hippocampal slices taken from a WT (**a**) and an ASIC1a KO (**b**) mice. The stimulation sites (Ch. 13 for a; Ch. 21 for **b**) are indicated by asterisks. Red and blue dots in (**a**,**b**) mark the two representative channels shown in (**c**) for LTD induction in WT (Ch. 18) and KO (Ch. 22) groups, respectively. (**c**) Time courses of normalized fEPSP slope for representative channels that exhibited LTD from the slices shown in (**a**,**b**). Inset: representative fEPSP traces at the time points indicated by the numbers. Calibration: 100 μV, 10 ms. (**d**) Averaged data for all activated channels that showed LTD in the slices shown in (**a**,**b**). (**e**) Time courses of mean fEPSP slope changes of all activated channels in 6 slices from 4 mice for each group. The mean fEPSP slope values during the baseline period were −30.3 μV/ms for WT and −33.3 μV/ms for KO group. (**f**) Summary data (number/slice, n = 6 slices/4 mice) of activated channels and LTD-showing channels in response to the LFS protocol in CA1 regions of WT and ASIC1a KO mice. (**g**) Time courses of mean fEPSP slope changes in response to bath application of DHPG (100 μM, 20 min) of all activated channels in 5 slices from 5 mice for each group. Inset: representative fEPSP traces at the time points indicated by the numbers. Calibration: 100 μV, 10 ms. The mean fEPSP slope values during the baseline period were −44.8 μV/ms for WT and −43.7 μV/ms for KO group. (**h**) Summary data (number/slice, n = 5 slices/5 mice) of activated channels and LTD-showing channels in response to DHPG application in CA1 regions of WT and ASIC1a KO mice. Horizontal bars denote the period of LFS delivery (**c–e**) or DHPG application (**g**). Error bars in (**e–h**) (many are smaller than the symbols) represent SEM.

LTD is another important form of synaptic plasticity in the central nervous system^15^,^28^. Therefore, we next investigated the effects of genetic deletion of ASIC1a on the inducibility of LTD in mouse hippocampus using two different protocols. First, we applied low frequency stimulations (LFS, 900 pulses, 1 Hz), a widely used classical protocol^29^, to induce electrical LTD. In WT slices, LFS caused depression of fEPSP in most activated channels lasting for 60 min (Fig. 3a,c). Similar results were obtained from the ASIC1a KO slices (Fig. 3b,c), suggesting that the deletion of ASIC1a has no effect on the induction of LTD by LFS in CA1 synapses. On average, the activated channels in the representative slices shown in Fig. 3a,b exhibited fEPSP slopes equivalent to 61.8% and 69.1% of baseline at 60 min after LFS for WT and ASIC1a KO groups, respectively (Fig. 3d). Pooled data for all slices recorded (6 slices from 4 WT and 6 slices from 4 ASIC1a KO mice) revealed similar levels of LTD induction by LFS between the two groups (Fig. 3e). The fEPSP slope was depressed to 58.4 ± 1.7% (*P* < 0.001 vs. baseline, paired t-test) and 62.9 ± 1.7% (*P* < 0.001 vs. baseline, paired t-test) of baseline at 60 min after LFS for WT and ASIC1a KO groups, respectively. The number of channels that developed LTD was 12.5 ± 1.6 out of a total of 15.2 ± 1.4 activated channels per slice, with an induction ratio of 80.8 ± 8.1% (Fig. 3f, n = 6 slices/4 mice) for WT hippocampal slices. For slices from ASIC1a KO mice, the number of channels that developed LTD was 10.5 ± 1.5 out of a total of 13.2 ± 0.9 activated channels per slice, with an induction ratio of 79.2 ± 9.3% (Fig. 3f, n = 6 slices/4 mice), which did not differ significantly from the values for the WT group.

Secondly, we induced chemical LTD through activation of group I metabotropic glutamate receptors by bath infusion of (RS)-3,5-dihydroxyphenylglycine (DHPG), which is another form of widely-studied LTD in the hippocampus^30^,^31^. As expected, bath application of 100 μM DHPG (for 20 min) to hippocampal slices from WT mice resulted in a persistent and stable LTD that lasted for at least 50 min (Fig. 3g). However, no appreciable difference was detected either in the magnitude or time course of DHPG-evoked LTD between WT and ASIC1a KO groups. The mean fEPSP slopes were 56.5 ± 1.5% and 59.6 ± 2.2% of baseline at 50 min after DHPG washout for slices prepared from WT and ASIC1a KO mice, respectively (Fig. 3g, n = 5 slices/5 mice, *P* < 0.001 vs. baseline, paired t-test, for both genotypes). For WT slices, the number of channels that showed DHPG-induced LTD was 13.2 ± 1.1 out of a total of 15.0 ± 1.1 activated channels per slice, with an induction ratio of 81.9 ± 5.0%; while for the ASIC1a KO slices, these values were 14.0 ± 3.7, 17.8 ± 3.2, and 76.7 ± 8.0%, respectively (Fig. 3h). Taken together, these results suggest that ASIC1a selectively regulates the inducibility of LTP in the hippocampal CA1 region, without affecting either electrical or chemical LTD.

### Pharmacological blockade of ASIC1a produces similar results as genetic deletion

**Figure 4:**
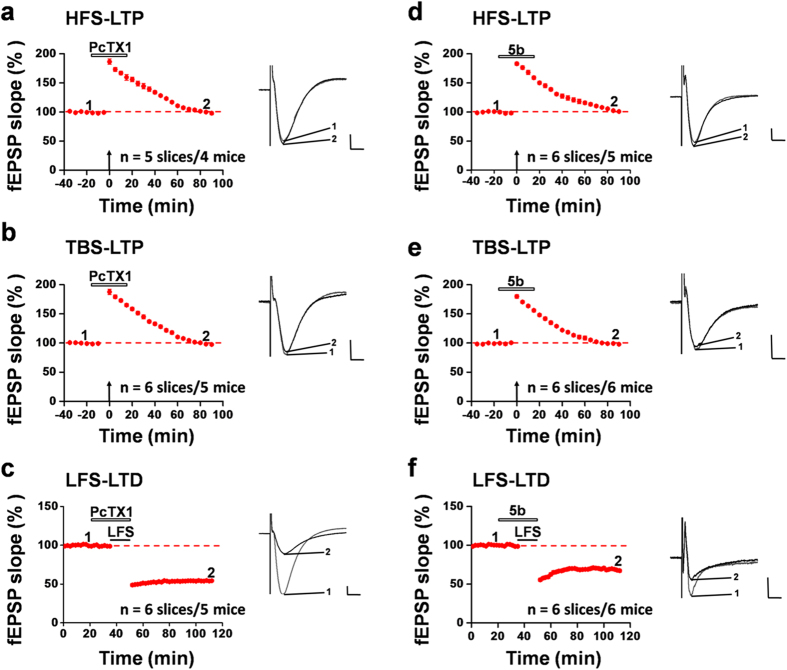
Pharmacological blockade of ASIC1a selectively inhbibits LTP without affecting LTD in the hippocampus. (**a**) Time course of mean fEPSP slope changes induced by HFS of all activated channels in 5 slices from 4 WT mice, treated with PcTX1 (100 nM) through bath application 15 min before HFS delivery and washed out at 15 min after HFS. (**b**) Similar to (**a**), but TBS was used to induce LTP. The mean data were from activated channels in 6 slices from 5 mice. (**c**) Similar to (**a**), but LFS was used to induce LTD. PcTX1 was bath applied 15 min prior to and during LFS. The mean data were from activated channels in 6 slices from 5 mice. (**d**) Similar to (**a**), but compound 5b (100 nM), a recently characterized potent inhibitor of ASIC1a, was bath applied 15 min before HFS delivery and washed out at 15 min after HFS. The mean data were from activated channels in 6 slices from 5 mice. (**e**) Similar to (**b**) but compound 5b was used in place of PcTX1. The mean data were from activated channels in 6 slices from 6 mice. (**f**) Similar to (**c**), but compound 5b was used in place of PcTX1. The mean data were from activated channels in 6 slices from 6 mice. Insets in a-f are representative fEPSP traces at the time points indicated by the numbers in the corresponding time course graphs. Calibration for all panels: 100 μV, 10 ms. The mean fEPSP slope values during the baseline period were −32.5 μV/ms, −42.3 μV/ms, −41.5 μV/ms, −44.8 μV/ms, −36.7 μV/ms and −47.5 μV/ms for (**a–f**), respectively. Arrows in (**a,b,d,e**) indicate the HFS or TBS application. Horizontal bars in (**a**–**f**) denote the period of PcTX1 or compound 5b or LFS application as indicated. Error bars (many are smaller than the symbols) represent SEM.

One of the common concerns of global gene ablation is the possibility of developmental compensation, which could confound the interpretation of the observed phenotype. Therefore, we employed a pharmacological approach by testing the effect of acute local application of a selective ASIC1a inhibitor, psalmotoxin 1 (PcTX1)^32^, on the synaptic plasticity in mouse hippocampus. Not surprisingly, pre-exposure of the hippocampal slice to PcTX1 (100 nM, 30 min) greatly reduced the success rate of HFS-evoked LTP induction. Among the 78 activated channels (15.6 ± 0.8 per slice) from 5 slices recorded, only 10 channels (2.0 ± 1.1 per slice) exhibited clear LTP (induction ratio: 13.3 ± 7.3%). Similarly, pre-treatment with the same dose of PcTX1 substantially inhibited the induction of LTP by TBS across the hippocampal network, with the induction ratio of 20.0 ± 4.2%. Overall, the mean fEPSP slope for activated channels in all PcTX1-treated slices was 98.6 ± 2.4% of baseline at 90 min after HFS (Fig. 4a, n = 5 slices/4 mice, *P* = 0.136 vs. baseline, paired t-test) and 98.2 ± 1.9% of baseline at 90 min after TBS (Fig. 4b, n = 6 slices/5 mice, *P* = 0.484 vs. baseline, paired t-test). In accordance with the data from the ASIC1a KO mice, bath application of PcTX1 (100 nM, added 15 min prior to and during the LFS) to the WT hippocampal slices had no effect on the probability of LTD induction by LFS. In total, 111 channels (18.5 ± 1.1 per slice) were activated and showed typical fEPSP in 6 slices analyzed, among which 94 channels (15.7 ± 1.6 per slice) underwent LTD in response to LFS, yielding an induction ratio of 84.5 ± 7.5% in the PcTX1-treated group, which is not statistically different from that of the control group (80.8 ± 8.1%). Pooled data from 6 slices/5 mice showed that PcTX1 affected neither the magnitude nor time course of LTD induction in the CA1 area (Fig. 4c, 54.3 ± 1.3% of baseline at 60 min after LFS, n = 6 slices/5 mice, *P* < 0.001 vs. baseline, paired t-test).

To further confirm the specificity of the PcTx1 effect, we used a structurally different ASIC1a inhibitor, 2-oxo-2H-chromene-3-carboxamidine derivative 5b (compound 5b), which was recently identified as an orthosteric antagonist of ASIC1a, with an apparent IC_50_ of 27 nM when measured at pH 6.7^33^. Acute application of compound 5b (100 nM, 30 min) to WT hippocampal slices decreased the probability of LTP induction by the HFS and TBS protocols in the CA1 region. Among the 78 activated channels (13.0 ± 1.3 per slice, 6 slices/5 mice) recorded, only 16 channels (2.7 ± 1.1 per slice) underwent clear LTP in response to the HFS delivery, with an induction ratio of 20.7 ± 7.8%. For the TBS-elicited LTP, the induction ratio was 23.6 ± 5.1% (n = 6 slices/6 mice) in the compound 5b-treated hippocampal slices. Summarizing all slices together, the mean fEPSP slope in the compound 5b-treated slices was 103.4 ± 2.6% of baseline at 90 min after HFS (Fig. 4d, n = 6 slices/5 mice, *P* = 0.959 vs. baseline, paired t-test) and 106.0 ± 1.4% of baseline at 90 min after TBS (Fig. 4e, n = 6 slices/6 mice, *P* = 0.819 vs. baseline, paired t-test). However, the LFS-induced LTD was not influenced by compound 5b. Neither the induction ratio (87.8 ± 2.9%) nor the magnitude of the electrical LTD was affected by compound 5b (Fig. 4f, fEPSP slope: 67.2 ± 2.0% of baseline at 60 min after LFS, n = 6 slices/6 mice, *P* < 0.001 vs. baseline, paired t-test). Thus, pharmacological blockade of ASIC1a function yielded similar results as genetic deletion of the ASIC1a gene.

### Expression of exogenous ASIC1a in the hippocampus of ASIC1a null mice rescues the LTP phenotype

**Figure 5:**
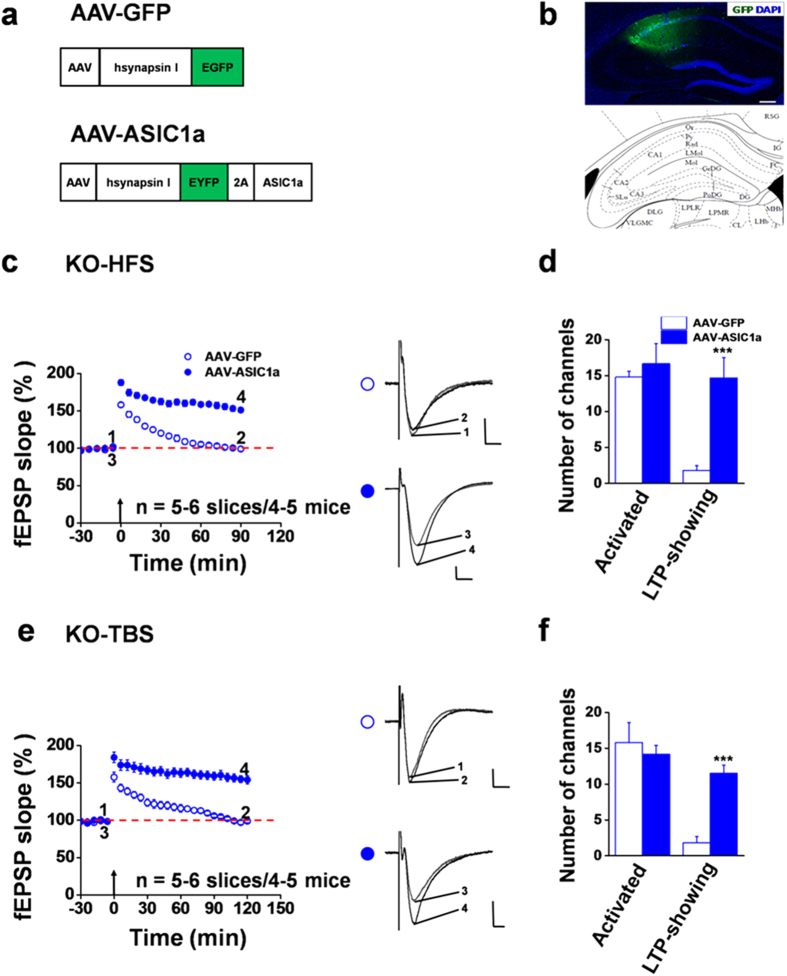
Expression of exogenous ASIC1a in the hippocampus rescues LTP induction in ASIC1a null mice. (**a**) Design of AAV viruses used for microinjection in the dorsal hippocampus. (**b**) Histological confirmation of the injection sites. The area with green fluorescence corresponds with the location of the injection needle. Scale bar: 200 μm. The brain atlas shown below was adapted from Paxinos and Franklin (2001). (**c**) Time courses of mean fEPSP slope changes in response to HFS of all activated channels in 5–6 slices from 4–5 ASIC1a KO mice injected with AAV-GFP (open blue circle) and AAV-ASIC1a (filled blue circle). Inset, representative fEPSP traces taken at the time points indicated by the numbers. Calibration: 100 μV, 10 ms. The mean fEPSP slope values during the baseline period were −42.5 μV/ms and −42.0 μV/ms for AAV-GFP and AAV-ASIC1a groups, respectively. (**d**) Summary data (number/slice, n = 5–6 slices/4–5 mice) of activated channels that responded to Schaffer collateral pathway stimulation and channels showing LTP in response to the HFS protocol in CA1 area of ASIC1a KO mice injected with AAV-GFP (open bars) and AAV-ASIC1a (filled bars). (**e**) Similar to (**c**), but TBS was used in place of HFS for LTP induction. Calibration: 100 μV, 10 ms. The mean fEPSP slope values during the baseline period were −37.1 μV/ms and −34.5 μV/ms for AAV-GFP and AAV-ASIC1a groups, respectively. (**f**) Summary data (number/slice, n = 5–6 slices/4–5 mice) of activated channels that responded to Schaffer collateral pathway stimulation and channels showing LTP in response to the TBS protocol in CA1 area of ASIC1a KO mice injected with AAV-GFP (open bars) and AAV-ASIC1a (filled bars). ***P < 0.001 for AAV-ASIC1a vs. AAV-GFP, unpaired t-test. Arrows in (**c,e**) indicate the HFS or TBS delivery. Error bars in (**c–f**) represent SEM.

To provide additional proof that the reduced probability of LTP induction in the ASIC1a null mice is indeed due to the loss of ASIC1a in the hippocampus, we performed rescue experiments by injecting an adeno-associated virus (AAV) vector carrying the full length ASIC1a cDNA (Fig. 5a) into the CA1 region of adult ASIC1a KO mice as previously described^10^. Using histological examinations, we confirmed that the exogenous proteins, as shown by the co-expressed enhanced yellow fluorescence protein (EYFP), were indeed expressed in the dorsal CA1 region of the injected mice (Fig. 5b). We found that virus-mediated expression of recombinant ASIC1a increased the probability of LTP induction by HFS. In 6 slices recorded, 88 (14.7 ± 2.8 per slice) channels exhibited LTP out of a total of 100 activated channels (16.7 ± 2.8 per slice), with an induction ratio of 87.5 ± 6.8% (Fig. 5d). However, the control virus, AAV-GFP, did not show such a rescue effect (induction ratio: 15.5 ± 5.5%, Fig. 5d, n = 5 slices/4 mice). The mean fEPSP slope for slices from AAV-ASIC1a-injected mice was 151.3 ± 3.4% of baseline at 90 min after HFS (n = 6 slices/5 mice, *P* < 0.001 vs. baseline, paired t-test), while that for slices from AAV-GFP-injected mice was 99.1 ± 3.1% of baseline (n = 5 slices/4 mice, *P* = 0.659 vs. baseline, paired t-test, Fig. 5c). Similarly, TBS-evoked LTP was also rescued by virus-mediated ASIC1a overexpression, with the induction ratio recovered to 81.0 ± 4.4% (n = 6 slices/4 mice), as compared with the control AAV-GFP group (11.1 ± 5.7%, n = 5 slices/5 mice, Fig. 5f). The mean fEPSP slope for the AAV-ASIC1a group was 154.2 ± 5.6% of baseline at 120 min after TBS (n = 6 slices/4 mice, *P* = 0.001 vs. baseline, paired t-test), while that for the AAV-GFP group was 98.9 ± 2.3% of baseline (n = 5 slices/5 mice, *P* = 0.153 vs. baseline, paired t-test, Fig. 5e). These results clearly demonstrate that ASIC1a is the key missing component in the hippocampal CA1 region of the global ASIC1a KO mice that regulates LTP induction in the majority of neuronal synapses.

### Enhancing NMDAR function partially rescues the impaired LTP induction in hippocampal CA1 region of ASIC1a null mice

**Figure 6:**
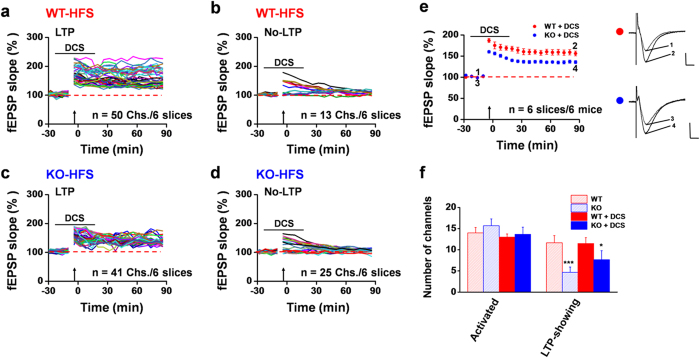
Partial restoration of impaired HFS-evoked LTP in hippocampal slices from ASIC1a KO mice by the NMDAR agonist DCS. (**a,b**) Pooled data for fEPSP slope changes of 50 LTP (**a**) and 13 No-LTP (**b**) channels in response to HFS in 6 slices from 6 WT mice treated with DCS (50 μM, 40 min). (c,d) Pooled data for fEPSP slope changes of 41 LTP (**c**) and 25 No-LTP (**d**) channels in response to HFS in 6 DCS-treated slices from 6 ASIC1a KO mice. (**e**) Time courses of mean fEPSP slope changes in response to HFS of all activated channels in 6 DCS-treated slices from 6 mice each for WT (red) and ASIC1a KO (blue) groups. Inset: representative fEPSP traces at the time points indicated by the numbers. Calibration: 100 μV, 10 ms. The mean fEPSP slope values during the baseline period were −41.4 μV/ms for WT and −36.4 μV/ms for KO group. (**f**) Summary data (number/slice, n = 6 slices/6 mice) of activated channels that responded to Schaffer collateral pathway stimulation and LTP channels that responded to HFS in the presence of DCS in the CA1 regions of WT (filled red bar) and ASIC1a KO (filled blue bar) mice. The data from Fig. 2h were re-plotted here for comparison (stripped red and blue bars). ***P < 0.001 for ASIC1a KO vs. WT; *P < 0.05 for ASIC1a KO+DCS vs. WT+DCS, unpaired t-test. Arrows in (**a–e**) indicate the HFS delivery. Horizontal bars in (**a–e**) denote the application of DCS. Error bars in (**e,f**) represent SEM.

Previous studies have suggested that ASIC1a may regulate hippocampal LTP through augmentation of NMDAR activation due to its role in causing membrane depolarization^6^. To examine the possible mechanisms underlying ASIC1a regulation of LTP inducibility, we tested whether enhancing NMDAR activity could rescue the impaired LTP seen in the ASIC1a null mice. To this end, we applied DCS, a well-known co-agonist of NMDAR that binds to the glycine site and enhances receptor activation in the presence of glutamate^24^,^34^. We first checked whether DCS (50 μM), applied at 20 min before HFS or TBS and washed out at 20 min after tetanus stimulation, could affect normal LTP induction in the hippocampus of the WT mice. As shown in Fig. 6a,b, in 6 slices/6 mice treated with DCS, 50 were LTP channels while 13 were No-LTP channels. These values did not differ significantly from the results obtained from naïve control slices shown in Fig. 1e,f. The mean fEPSP slope remained at 156.2 ± 5.8% of baseline at 90 min after HFS in the DCS-treated slices (Fig. 6e, n = 6 slices/6 mice, *P* = 0.002 vs. baseline, paired t-test), indicating that DCS alone has no major effect on the inducibility of hippocampal LTP under normal conditions.

We next asked whether DCS treatment could rescue the LTP deficit in the hippocampal slices taken from ASIC1a KO mice. Interestingly, treatment of slices from ASIC1a KO mice with DCS increased the number of LTP channels in response to HFS, but not to the extent achieved by the WT control slices. Among all activated channels (13.7 ± 1.7 per slice) recorded from 6 slices/6 mice, 41 (7.7 ± 2.1 per slice) were LTP channels while the remaining 25 were No-LTP channels (Fig. 6c,d). This yielded an induction ratio of 59.4 ± 8.2% (Fig. 6f), which is significantly higher than that of the untreated ASIC1a KO group (Fig. 2h, 29.8 ± 7.7%, *P* = 0.026 vs. ASIC1a KO-DCS, unpaired t test), but still lower than the WT group either untreated (Fig. 2h, 82.5 ± 7.4%, *P* = 0.003 vs. ASIC1a KO-DCS, unpaired t test) or treated with DCS (Fig. 6f, 79.3 ± 6.8%, *P* = 0.036 vs. ASIC1a KO-DCS, unpaired t test). The mean fEPSP slope reached 135.8 ± 3.4% of baseline at 90 min after HFS for the DCS-treated ASIC1a KO group (Fig. 6e, n = 6 slices/5 mice, *P* = 0.031 vs. baseline, Wilcoxon Signed Rank test), which is higher than the untreated ASIC1a KO group (106.4 ± 2.8% of baseline, *P* = 0.002 vs. ASIC1a KO-DCS, unpaired t test), but lower than the DCS-treated WT group (156.2 ± 5.8% of baseline, *P* = 0.037 vs. ASIC1a KO-DCS, unpaired t test). Similar results were obtained when the TBS protocol was used (WT vs. ASIC1a KO: induction ratio, 90.2 ± 4.5% vs. 75.6 ± 12.8%; LTP magnitude, 156.3 ± 5.2% vs. 139.7 ± 3.3% of baseline at 120 min after TBS; Supplementary Fig. S4).

**Figure 7:**
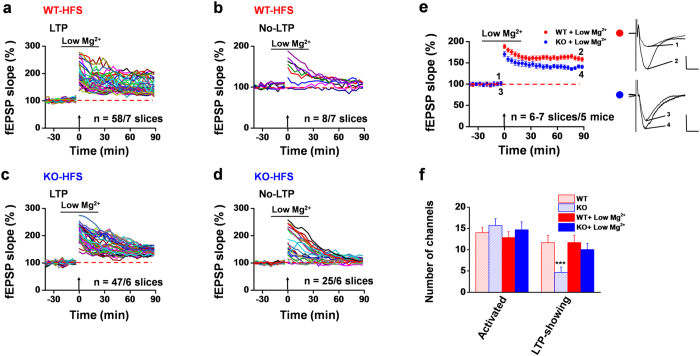
Partial restoration of impaired HFS-evoked LTP in hippocampal slices from ASIC1a KO mice by low Mg^2+^ treatment. (**a,b**) Pooled data for fEPSP slope changes of 58 LTP (**a**) and 8 No-LTP (**b**) channels in response to HFS in 7 slices from 5 WT mice treated with low Mg^2+^ ACSF (0.1 mM, 40 min). (**c,d**) Pooled data for fEPSP slope changes of 47 LTP (**c**) and 25 No-LTP (**d**) channels in response to HFS in 6 low Mg^2+^-treated slices from 5 ASIC1a KO mice. (**e**) Time courses of mean fEPSP slope changes in response to HFS of all activated channels in 6–7 low Mg^2+^-treated slices from 5 mice each for WT (red) and ASIC1a KO (blue) groups. Inset: representative fEPSP traces at the time points indicated by the numbers. Calibration: upper, 100 μV, 10 ms; lower, 50 μV, 10 ms. The mean fEPSP slope values during the baseline period were −41.4 μV/ms for WT and −40.9 μV/ms for KO group. (**f**) Summary data (number/slice, n = 6–7 slices/5 mice) of activated channels that responded to Schaffer collateral pathway stimulation and LTP channels that responded to HFS in the low Mg^2+^ ACSF in the CA1 regions of WT (filled red bar) and ASIC1a KO (filled blue bar) mice. The data from Fig. 2h were re-plotted here for comparison (stripped red and blue bars). ***P < 0.001 for ASIC1a KO vs. WT, unpaired t-test. Arrows in (**a–e**) indicate the HFS delivery. Horizontal bars in (**a–e**) denote the application of low Mg^2+^ ACSF. Error bars in (**e,f**) represent SEM.

To exclude the possibility that the incomplete rescue of impaired LTP resulted from the low dose of DCS used, we applied a higher concentration of DCS (100 μM) for the same duration. However, the impaired HFS-evoked LTP was still not fully restored to the WT level (WT vs. ASIC1a KO: induction ratio, 87.4 ± 4.9% vs. 63.3 ± 6.8%; LTP magnitude, 158.7 ± 5.3% vs. 139.5 ± 6.0% of baseline at 90 min after HFS; Supplementary Fig. S5). Furthermore, given that DCS is a co-agonist of NMDAR, it might be inherently unable to fully activate NMDAR. As an alternative way to enhance NMDAR function, we lowered Mg^2+^ concentration in the artificial cerebrospinal fluid (ACSF) to 0.1 mM and treated the slices with the low Mg^2+^ ACSF from 20 min before till 20 min after HFS. The low Mg^2+^ treatment is believed to fully activate NMDARs under our experimental conditions. However, similar to DCS, we found that the low Mg^2+^ ACSF only partially, but not completely, rescued the impaired HFS-induced LTP in the hippocampal slices from ASIC1a KO mice (Fig. 7). In a total of 6 slices analyzed, 47 activated channels exhibited LTP, while 25 channels failed to show LTP, with the induction ratio of 70.4 ± 2.2% (Fig. 7c,d,f). On the other hand, the low Mg2^+^ treatment did not affect LTP inducibility in slices from the WT mice, yielding 58 LTP channels and 8 No-LTP channels in 7 slices from 5 mice (Fig. 7a,b). The induction ratio was 89.3 ± 5.2%, which is not statistically different from untreated WT slices (82.5 ± 7.4%, Fig. 2h). Similarly, the LTP magnitude at 90 min after HFS of the low Mg^2+^-treated ASIC1a KO group was still significantly lower than that of the WT group (WT vs. ASIC1a KO, 158.5 ± 4.9% vs. 140.7 ± 4.0%, Fig. 7e). These results, together with the DCS data, suggest that both NMDAR-dependent and NMDAR-independent mechanisms are involved in ASIC1a regulation of hippocampal synaptic plasticity.

The failure of DCS and low Mg^2+^ treatment to fully rescue the impaired hippocampal LTP induction in ASIC1a KO mice could be attributed to a change in postsynaptic NMDAR expression in the ASIC1a null mice. To address this possibility, we examined the expression levels of several glutamate receptor subunits in the postsynaptic density (PSD) fraction of the hippocampus. As shown by the western blot analysis (Supplementary Figs S6 and S7), no alteration in the amount of several NMDAR and AMPA receptor subunits was detected in samples prepared from the ASIC1a KO mice as compared with WT mice. Therefore, the impairment of hippocampal LTP induction and the lack of its full restoration by enhancing NMDAR function in ASIC1a null mice represent a functional deficit, rather than alterations in ionotropic glutamate receptor expression.

## Discussion

ASIC1a has been known to play important roles in a variety of physiological functions in the brain, including learning/memory^6^, fear^8^,^19^, anxiety^9^,^35^ and pain perception^36^,^37^. However, the synaptic basis for ASIC1a to affect the above processes remains unclear. Although it has been suggested that ASIC1a may contribute to certain forms of synaptic plasticity, few reported studies have addressed this issue and the results are controversial^20^. Here, we evaluated the role of ASIC1a in five forms of hippocampal long-term plasticity using a multi-electrode array recording system. We show that gene ablation and pharmacological blockade of ASIC1a partially, but not completely, abolished LTP induction in mouse CA1 region, without an effect on hippocampal LTD. We also found that enhancing NMDAR function only partially restored LTP inducibility in the ASIC1a null hippocampus, indicating that ASIC1a contributes to hippocampal synaptic plasticity through multiple mechanisms, including both NMDAR-dependent and -independent ones.

It is well known that the inducibility of synaptic plasticity is governed by cellular and molecular processes which are stochastic in nature. In other words, not all synapses undergo LTP/LTD even under identical induction conditions^38^. Conventional single electrode recording techniques typically could not reveal this heterogeneity due to the intrinsic bias of the recording procedures. In this regard, the multi-electrode array recording provides a novel and unbiased approach to address the probabilistic nature of LTP or LTD induction at multiple sites both simultaneously and objectively^39^,^40^,^41^. In addition, compared with other electrophysiological approaches, multi-electrode array enables a much longer time of LTP or LTD monitoring (several hours or even days)^42^,^43^. Thus, introduction of multi-site electrophysiology may bring in new insights into the synaptic plasticity field by providing more complete and undistorted information on the induction properties and spatial distribution of LTP and/or LTD^23^.

Here, we employed a 64-channel multi-electrode array recording system to study synaptic plasticity in hippocampal slices from WT and ASIC1a KO mice. As expected, this system revealed a probabilistic nature of LTP induction across a broad area of the CA1 region when the Schaffer collateral pathway was subjected to tetanic stimulation at a single site. In WT mice, only ~85% of activated channels, i.e. those that displayed fEPSP, developed LTP in response to HFS or TBS. A similar success rate was also obtained for LTD induced by LFS or DHPG. The lower than 100% success rates may have arisen from two reasons. First, it may be ascribed to the variance in the expression levels of critical LTP- or LTD-inducing receptors (e.g. NMDARs) or the intrinsic excitability among individual sites^38^. Second, to ensure detection of LTP/LTD in conventional single electrode recordings, the stimulation strength is typically adjusted to elicit 40–60% of the maximal EPSP or EPSC at an individual site. In the MED64 system, however, the field stimulation to all areas is provided solely from one channel, which cannot guarantee that all activated channels receive the optimal stimulation intensity needed to develop LTP or LTD, despite the careful selection of the stimulation intensity based on the input-output curve that represents the mean of all activated channels in the slice. Therefore, some channels might have received too weak stimulation that was below the threshold of LTP induction whereas a few others might have received too strong stimulation that occluded LTP development. Regardless of the causes, the ~15% No-LTP channels found in WT mice demonstrated that in the presence of ASIC1a, the large majority of neurons are able to develop LTP under our recording conditions.

Our results show that genetic ablation of ASIC1a did not affect the baseline synaptic property, but strongly reduced (although not fully abolished) the probability of LTP induction by either HFS or TBS. The critical involvement of ASIC1a in regulating hippocampal LTP induction was further confirmed by pharmacological and virus-mediated rescue experiments. Because of the objective and broad coverage of neurons in the CA1 region by the multi-electrode array system, our findings may help reconcile some of the previous controversies regarding the role of ASIC1a in synaptic plasticity^6^,^44^. Specifically, our unbiased recordings clearly show that at least for some neurons, ASIC1a is not absolutely required for LTP induction. Under our experimental conditions, ~13–30% of activated channels in the CA1 region of ASIC1a null mice or ASIC1a antagonist-treated WT mice still exhibited LTP in response to HFS or TBS. With a stronger LTP protocol, this ratio was increased to ~53%. These observations can be best explained if ASIC1a acts by reducing the threshold for LTP induction in the hippocampal neurons. Hence, the strength of LTP induction may strongly influence the outcomes of single electrode recordings performed on ASIC1a KO animals, leading to the conclusion that ASIC1a was involved^6^ or not involved^44^ in LTP induction. However, because of the intrinsic heterogeneity of hippocampal neurons, the expression levels of ASIC1a^45^ and other key contributors to LTP can vary markedly among different neuronal populations. Therefore, the possible presence of an ASIC1a-independent population(s) of CA1 neurons for LTP induction, albeit very small, cannot be ruled out. Moreover, there could also be a separate neuronal population(s) that absolutely requires ASIC1a for LTP induction, since even a stronger HFS protocol still could not make the KO slices achieve the LTP induction ratio to that of the WT slices. In such a case, the different targeting strategies (Nestin-Cre conditional vs. global KO) used between the previous studies could also be responsible for the conflicting results observed based on single electrode recordings.

LTD represents another important form of synaptic plasticity in the brain^15^,^28^. To our knowledge, however, no study has been reported on the role of ASIC1a in LTD. In the present study, we sought for the first time to address this question in the hippocampus. However, genetic ablation or pharmacological blockade of ASIC1a affected neither the magnitude nor time course of LTD induction through either electrical stimulation or a chemical method. The exact reasons for this selective role of ASIC1a in hippocampal LTP but not LTD are unclear. Possibly, ASIC1a is specifically involved in distinct LTP-related signaling pathways, which exert a minor effect on LTD. It is also possible that the LTD protocols used in the current study preferentially targeted the cell types that express no or low levels of ASIC1a in the hippocampus^45^.

For the mechanisms underlying the involvement of ASIC1a in hippocampal LTP, previous studies have consistently implicated ASIC1a-induced membrane depolarization as a major route to modulate NMDAR activation^6^,^33^, through which synaptic plasticity occurs. If this were true, then enhancing NMDAR function would be expected to overcome the inhibitory effect of ASIC1a gene ablation on LTP inducibility. Indeed, we show that augmentation of NMDAR activities in slices from ASIC1a null mice by DCS, an NMDAR co-agonist, or the low Mg^2+^ ACSF, partially restored the induction ratio of LTP in the CA1 region. However, despite the strong conditions used to facilitate NMDAR activation, i.e. 100 μM DCS or 0.1 mM Mg^2+^, the rescue was incomplete, suggesting that both NMDAR-dependent and –independent mechanisms may be involved in ASIC1a regulation of hippocampal synaptic plasticity. Apart from NMDAR, voltage-gated calcium channels, calcium-permeable AMPA receptors (CP-AMPARs), and even protein kinases are also key players of LTP induction, and ASIC1a could act at any of those. Interestingly, it was recently reported that ASIC1a activation is both necessary and sufficient to mediate the increased CP-AMPAR activity that occurs following ischemia or acidosis^46^. CP-AMPARs are known to play an important role in hippocampal LTP^47^,^48^,^49^. Therefore, the exact nature of the non-NMDAR component(s) and the underlying mechanism by which ASIC1a modulates its function are interesting questions that warrant future investigations.

In summary, we addressed the role of ASIC1a in multiple forms of long-term synaptic plasticity in the hippocampus with an *in vitro* multi-channel recording system. We demonstrate that ASIC1a critically contributes to the probability of induction of hippocampal LTP but not LTD through both NMDAR-dependent and NMDAR-independent mechanisms. Our findings highlight the great potential of harnessing multi-electrode electrophysiology to study contributions of key molecular components to synaptic plasticity in the brain.

## Methods

### Animals

The experiments were performed on C57BL/6J WT or ASIC1a KO (with congenic C57BL/6J background) mice (6–10 week old)^37^,^50^. All animals were housed in groups of three to four per cage under standard laboratory conditions (12 hr light/12 hr dark, temperature 22–26 °C, air humidity 55–60%) with ad libitum water and mouse chow. All animal procedures were carried out in accordance with the guidelines for the Care and Use of Laboratory Animals of Shanghai Jiao Tong University School of Medicine and approved by the Institutional Animal Care and Use Committee (Department of Laboratory Animal Science, Shanghai Jiao Tong University School of Medicine) (Policy Number DLAS-MP-ANIM. 01–05).

### Preparation of hippocampal slices

The general procedures for making the acute hippocampal slices are similar to those described previously^21^. Briefly, the mouse was anesthetized and decapitated. The whole brain was rapidly removed and immersed into a cold bath of oxygenated (equilibrated with 95% O_2_ and 5% CO_2_) ACSF containing (in mM): NaCl 124, KCl 2.5, NaH_2_PO_4_ 1.0, MgSO_4_ 1, CaCl_2_ 2, NaHCO_3_ 25 and glucose 10, pH 7.35–7.45. Three transverse hippocampal slices (300 μm thick) were cut with a vibrating tissue slicer (Leika, VT1000S, Germany) and transferred to an incubation chamber with oxygenated ACSF at 31 °C. Slices were allowed to recover for at least 2 hr before electrophysiological recording was attempted.

### Multi-channel field potential recordings

A commercial 64-channel recording system (MED64, Panasonic Alpha-Med Sciences, Japan) was used for extracellular field potential recordings. Procedures for preparation of the MED64 probe (P515A, Panasonic, Japan) and multi-channel field potential recordings followed those described previously^39^,^40^,^41^. The MED64 probe had an array of 64 planar microelectrodes, each 50 × 50 μm in size, arranged in an 8 × 8 pattern (inter-electrode distance: 150 μm). Before use, the surface of the MED64 probe was treated with 0.1% polyethyleneimine (Sigma, MO, USA) in 25 mM borate buffer (pH 8.4) overnight at room temperature (22–23 °C). For recording, one slice was positioned on the MED64 probe in such a way that the hippocampal CA1 area was entirely covered by the recording dish mounted on the stage of an inverted microscope (IX51, Olympus, Japan). The slice was continuously perfused with oxygenated fresh ACSF at the rate of 2–3 ml/min with the aid of a peristaltic pump (Minipuls 3, Gilson, WI, USA). The perfusate temperature was maintained at 31 °C by an electronic heat control system (ThermoClamp-1, Automate Scientific, CA, USA) throughout the entire experimental period.

After a 10–15 min recovery period, one of the 64 available planar microelectrodes was selected for stimulation by visual observation through a charge-coupled device (CCD) camera connected to the inverted microscope. For test stimulation, monopolar, biphasic constant current pulses (0.1 ms in duration) generated by the data acquisition software (Mobius, Panasonic Alpha-Med Sciences) were applied to the Schaffer collateral pathway at 60 s intervals. The fEPSPs evoked at the CA1 area were amplified by a 64-channel amplifier, displayed on the monitor screen and stored on the hard disk of a microcomputer for off-line analysis. After selecting the best stimulation site, an input-output curve was first determined for each group using the measurements of fEPSP slopes or the number of activated channels (output) in response to a series of ascending stimulation intensities from 8 to 20 μA in 2 μA steps (input). Baseline synaptic responses were then stabilized for at least 30 min before any conditioning stimulation. The test stimulation intensity was set to be able to induce, on average, 30–60% of the maximal synaptic response according to the input-output curves. For LTP induction, a HFS protocol (100 Hz, 1s), a 4 × HFS protocol (100 Hz, 1s, repeated 4 times at 20 s intervals) or a TBS protocol (10 bursts repeated at 200 ms intervals, with four pulses at 100 Hz for each burst) was given at the same stimulation intensity as the baseline^21^. For LTD induction, a classical LFS protocol (1 Hz, 900 pulses) was used as described previously^40^,^41^. In this study, we also investigated the possibility of chemical LTD induction by bath perfusion of the group I metabotropic glutamate receptor agonist DHPG (100 μM, 20 min) as described previously^30^,^40^,^51^. After delivering the above protocols, the time course of fEPSP slope changes was monitored for 50 min to 2 hr according to the need of individual experiments.

To evaluate the acute effect of pharmacological inhibition of ASIC1a on hippocampal synaptic plasticity, we used the ASIC1a inhibitor PcTX1^32^ and compound 5b^33^. PcTX1 (100 nM) or compound 5b (100 nM) was given 15 min prior to HFS or TBS delivery and washed out 15 min after conditioning stimulation. For LTD, the same concentration of PcTX1 or compound 5b was applied 15 min before LFS and maintained continuously during LTD induction. To measure paired-pulse facilitation, paired-pulses to the Schaffer collateral pathway were delivered at intervals of 25, 50, 75, 100, and 200 ms and the paired-pulse ratio of the fEPSP slope of the second response to that of the first response was calculated and averaged. To determine the NMDAR dependence underlying the involvement of ASIC1a in hippocampal LTP inducibility, we bath applied DCS (50 μM or 100 μM, 40 min) or the low Mg^2+^ (0.1 mM) ACSF from 20 min prior to HFS or TBS delivery till 20 min after the conditioning, to see whether enhancing the activity of NMDAR could rescue the impaired LTP in the ASIC1a KO mice. The drug dose or Mg^2+^ concentration was chosen based on previous reports^6^,^52^ and our preliminary data.

### Virus preparation and injection

To express exogenous ASIC1a in the hippocampal CA1 area of ASIC1a KO mice, AAV vectors carrying the full length of ASIC1a cDNA were constructed. Specifically, the coding sequence for EYFP protein was fused to that for the N-terminus of mASIC1a by using a “self-cleaving” 2A-peptide. The cDNA for the fusion protein EYFP-2A-mASIC1a was subcloned to the pAAV-MCS vector and driven by the human synapsin I (hsynapsin I) promoter in order to specifically limit expression to neurons. This construct was then packaged into AAV2/9 chimeric virus with AAV9 capsids and AAV2 ITR (inverted terminal repeat) elements. The control AAV vector (AAV-GFP) was constructed by sub-cloning the EGFP coding sequence to the pAAV-MCS vector following the hsynapsin I promoter.

For virus injection, mice at the age of 6–8 weeks were anesthetized and placed in a stereotaxic frame (RWD Life Science, China). Virus (0.5 μl per side) was infused bilaterally into the dorsal hippocampus using a microelectrode connected with a microinjector pump (KDS 310, KD Scientific, USA) at a rate of 0.1 μl/min. Microelectrodes were left *in situ* for an additional 10 min to allow the virus to diffuse. The injection coordinates were as follows (relative to bregma): anteroposterior, −2.0 mm; lateral, ±1.6 mm; ventral, 1.6 mm from pial surface^53^. Mice were allowed to recover for at least 4 weeks before electrophysiological analysis. The injection sites were confirmed post-mortem by direct observation under an epifluorescence microscope (IX71, Olympus). Correctly targeted injections were defined as having fluorescence above background within the hippocampal CA1 area. Off-target injections were excluded from further analysis.

### Subcellular fractionation and western blotting

The purification of the PSD fraction was performed based on previous studies^54^,^55^ with some modifications. Briefly, the hippocampal tissue combined from 2 mice (WT or ASIC1a KO) was homogenized in a buffer containing 5 mM HEPES (pH 7.4), 1 mM MgCl_2_ and 0.5 mM CaCl_2_ in the presence of protease inhibitors. The homogenates were centrifuged at 1400 g for 10 min at 4 °C. The resulting supernatant (S1) was saved and the pellet was resuspended and centrifuged at 700 g resulting the supernatant (S1’). S1’ was added to S1 and centrifuged at 13,800 g for 10 min to obtain a crude membrane fraction (P2 fraction). The P2 fraction was resuspended in 0.32 M sucrose and loaded onto a discontinuous sucrose gradient (from top, 0.85 M: 1.0 M: 1.2 M = 3 ml: 3 ml: 3 ml), and then centrifuged for 2 hr at 82,500 g in an SW-41 rotor (Beckman Coulter). The synaptosome fraction between 1 M and 1.2 M sucrose was collected with a syringe needle and resuspended in a buffer containing 6 mM Tris (pH 8.1) and 0.5% Triton X-100. After 15 min treatment by Triton X-100, the suspension was centrifuged at 201,800 g (Beckman Coulter) for 1 hr and the final pellet (PSD) was dissolved using a buffer containing 0.2% SDS and protease inhibitors.

Protein samples were separated by SDS-PAGE and transferred to polyvinylidene difluoride filters. The filters were incubated overnight at 4 °C with appropriate antibodies. Secondary antibodies conjugated to horseradish peroxidase were added to the filters and then visualized in ECL solution. The visualization was performed via the ImageQuant LAS 4000 mini Molecular Imaging System (GE Healthcare Life Sciences), and the Image J software (NIH) was used for the analysis of band intensity. Antibodies used were as follows: ASIC1a (1:500; Santa Cruz), β-actin (1:1000; Chemicon), GluA1 (1:1000; Epitomics), GluA2 (1:1000; Millipore), GluN1 (1:1000; Epitomics), GluN2A (1:1000; Millipore), GluN2B (1:1000; Chemicon), PSD-95 (1:1000; Epitomics).

### Drugs

The drugs used in this study include: DHPG, PcTX1, compound 5b and DCS. All of these drugs were dissolved in distilled water as stock solutions and stored in small frozen aliquots at −20 °C. The stock solutions were diluted to the final desired concentration in ACSF immediately before use. DHPG was obtained from Tocris Cookson (Bristol, UK); PcTX1 was purchased from Peptide Institute (Osaka, Japan); DCS was from Sigma. Compound 5b was made and used as described in our recent paper^33^.

### Data analysis

All multi-channel electrophysiological data were analyzed off-line by the MED64 Mobius software. For quantification of the input-output relationship, the slope of fEPSP was measured and plotted as a function of stimulation intensities. The number of activated channels evoked at different stimulation intensities was also counted in a blind manner. For quantification of LTP and LTD data, the initial slope of fEPSP was measured, normalized and expressed as percentage change from the baseline level. Furthermore, the number of activated channels (i.e. with the fEPSP amplitude higher than −20 μV) vs. LTP-showing (fEPSP slope increased by at least 20% of baseline) or LTD-showing (fEPSP slope depressed by at least 15% of baseline) channels was counted and the induction ratio of LTP/LTD was calculated as follows: number of LTP/LTD-occurring channels/number of all activated channels ×100%^39^,^40^,^51^,^56^. All data are presented as mean ± S.E.M. When necessary, the statistical significance was assessed by paired or unpaired Student’s t test or Wilcoxon Signed Rank test using the Sigma Plot11.0 software. *P* < 0.05 was considered statistically significant.

## Additional Information

**How to cite this article**: Liu, M.-G. *et al.* Acid-sensing ion channel 1a contributes to hippocampal LTP inducibility through multiple mechanisms. *Sci. Rep.* **6**, 23350; doi: 10.1038/srep23350 (2016).

## Supplementary Material

Supplementary Information
