# Supplementary material for: Acid-sensing ion channel 1a contributes to hippocampal LTP inducibility through multiple mechanisms
Source: Sci Rep. 2016 Mar 21;6:23350. doi: 10.1038/srep23350 (PMC4800407; doi:10.1038/srep23350)
Supplement: Supplementary Information [file srep23350-s1.pdf]

Supplemental information

Acid-sensing ion channel 1a contributes to hippocampal LTP inducibility through multiple mechanisms

Ming-Gang Liu, Hu-Song Li, Wei-Guang Li, Yan-Jiao Wu, Shi-Ning Deng, Chen Huang, Oleksandr Maximyuk, Volodymyr Sukach, Oleg Krishtal, Michael X. Zhu and Tian-Le Xu

Supplementary Figures

Supplementaryl Figure S1

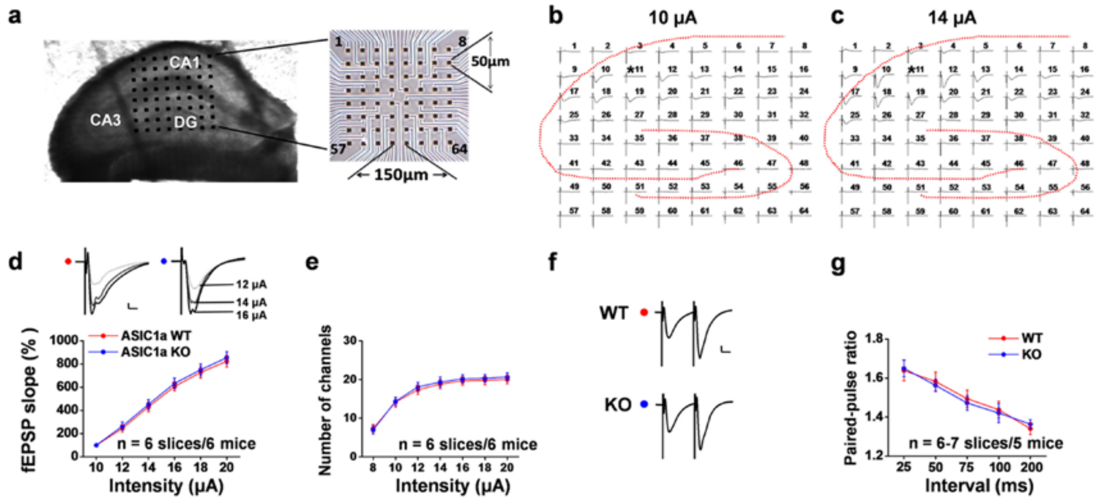

Supplementary Figure S1. Genetic deletion of ASIC1a does not alter basal synaptic transmission in the hippocampus

(a) Light microscopy photograph showing the location of the MED64 probe relative to the hippocampal slice (left) and the arrangement of the 8 × 8 recording array (inter-electrode distance, 150 µm; electrode size, 50 × 50 µm; right). (b,c) An overview of the multisite synaptic responses evoked in the CA1 region by delivering an electrical stimulation at 10 µA (b) and 14 µA (c). Asterisks indicate the stimulating channel (Ch. 11). Red dotted lines demarcate the contour of the hippocampus. (d,e) Input–output relationship of the fEPSP slope (d) or number of activated channels (e) in response to a series of graded stimulation intensities ranging from 8 to 20 µA at 2 µA steps. Shown are pooled data from WT (red) and ASIC1a KO (blue) mice (n = 6 slices/6 mice for each group). Example traces in response to 12 µA, 14 µA and 16 µA stimulations are illustrated at the top of (d) for the two groups. Calibration: 100 µV, 10 ms. (f) Representative traces of paired-pulse facilitation with an interval of 25 ms recorded in slices from WT (top) and ASIC1a KO mice (bottom). Calibration: 100 µV, 10 ms. (g) Paired-pulse ratio (slope of fEPSP2/slope of fEPSP1) recorded with intervals of 25, 50, 75, 100, and 200 ms in the CA1 region (n = 6-7 slices/5 mice). Error bars in (d, e, g) represent SEM.

Supplementary Figure S2

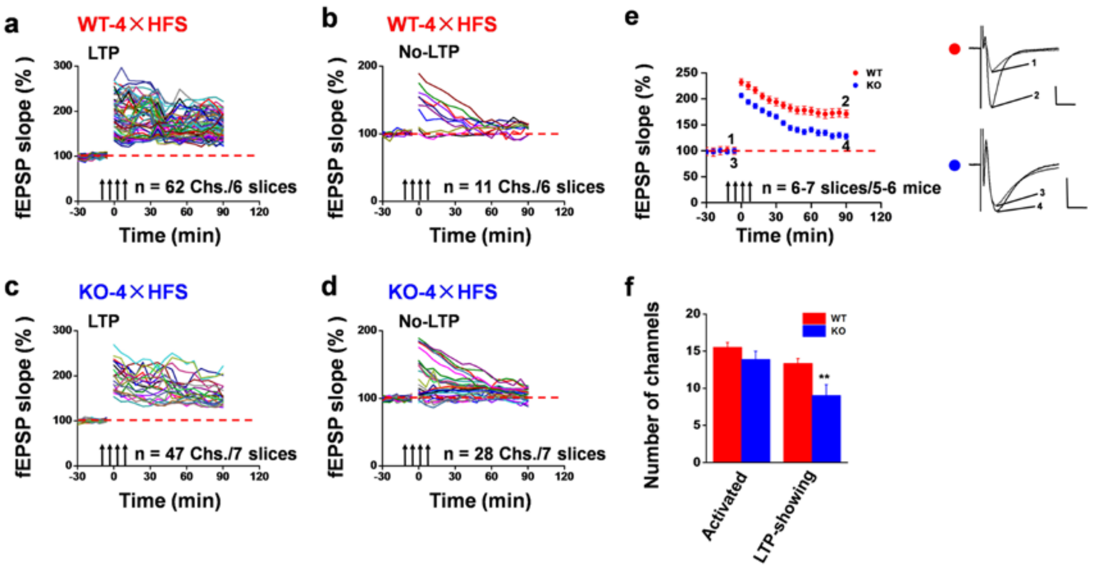

Supplementary Figure S2. Genetic deletion of ASIC1a reduces the probability of LTP induction by 4 × HFS in the hippocampus

(a,b) Pooled data for fEPSP slope changes of 62 LTP channels (a) and 11 No-LTP channels (b) that received 4 × HFS from 6 slices analyzed for 5 WT mice. (c,d) Pooled data for fEPSP slope changes of 47 LTP channels (c) and 28 No-LTP channels (d) that received 4 × HFS from 7 slices analyzed for 6 ASIC1a KO mice. ASIC1a KO mice still showed a deficit in LTP inducibility compared to WT mice even with the use of a stronger HFS protocol. (e) Time courses of mean fEPSP slope changes of all activated channels in 6-7 slices from 5-6 mice for WT (red) and ASIC1a KO (blue) groups. Inset: representative fEPSP traces at the time points indicated by the numbers. Calibration: upper, 100 μV, 10 ms; lower, 50 μV, 10 ms. The mean fEPSP slope values during the baseline period were -36.5 μV/ms for WT and -38.7 μV/ms for KO group. (f) Summary data (number/slice, n = 6-7 slices/5-6 mice) of activated channels that responded to Schaffer collateral pathway stimulation and LTP channels that responded to the 4 × HFS protocol in CA1 regions of WT and ASIC1a KO mice. ***P* < 0.01 for ASIC1a KO vs. WT, unpaired Student's t-test. Arrows in (a-e) indicate the 4 × HFS delivery (not in proportion). Error bars in (e,f) represent SEM.

Supplementary Figure S3

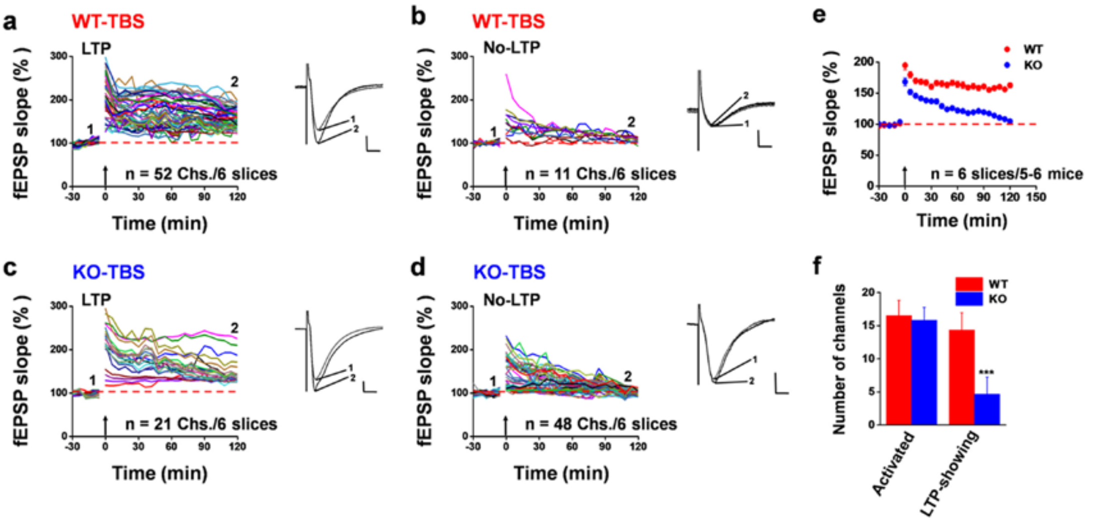

Supplementary Figure S3. Genetic deletion of ASIC1a reduces the probability of LTP induction by TBS in the hippocampus

(a,b) Pooled data for fEPSP slope changes of 52 LTP channels (a) and 11 No-LTP channels (b) that received TBS from 6 slices analyzed for 6 WT mice. (c,d) Pooled data for fEPSP slope changes of 21 LTP channels (c) and 48 No-LTP channels (d) that received TBS from 6 slices analyzed for 5 ASIC1a KO mice. While the WT displayed more LTP than No-LTP channels, ASIC1a KO had more No-LTP than LTP channels in response to the TBS protocol. Insets in (a-d) show representative fEPSP traces at the time points indicated by the numbers. Calibration: (a,c,d), 100 μV, 10 ms; (b), 25 μV, 10 ms. (e) Time courses of mean fEPSP slope changes of all activated channels in 6 slices from 5-6 mice for WT (red) and ASIC1a KO (blue) groups. The mean fEPSP slope values during the baseline period were -41.5 μV/ms for WT and -43.6 μV/ms for KO group. (f) Summary data (number/slice, n = 6 slices/5-6 mice) of activated channels that responded to Schaffer collateral pathway stimulation and LTP channels that responded to the TBS protocol in CA1 regions of WT and ASIC1a KO mice. ***P < 0.001 for ASIC1a KO vs. WT, unpaired Student's t-test. Arrows in (a-e) indicate the TBS delivery. Error bars in (e,f) represent SEM.

Supplementary Figure S4

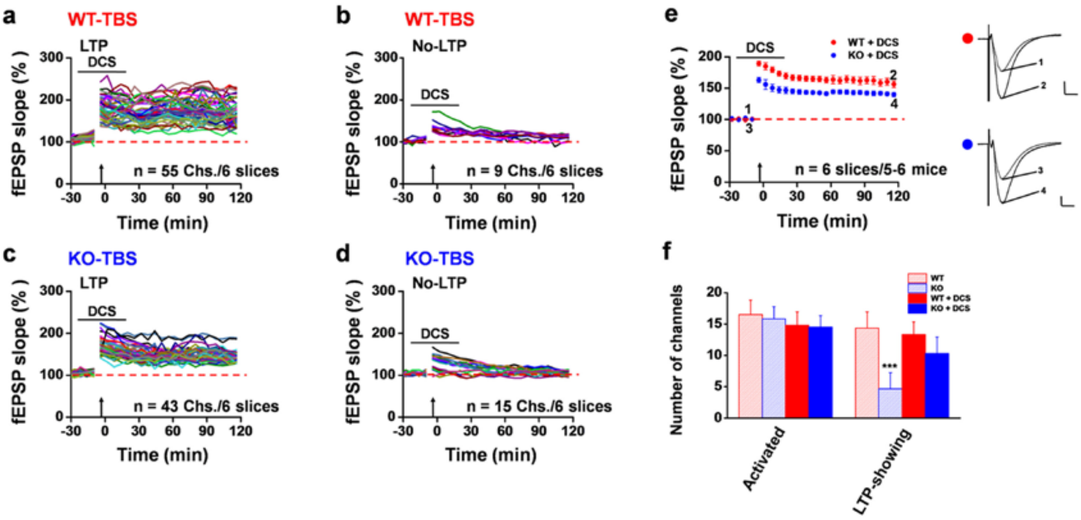

Supplementary Figure S4. Partial restoration of impaired TBS-evoked LTP in hippocampal slices from ASIC1a KO mice by enhancing NMDAR function with DCS

(a,b) Pooled data for fEPSP slope changes of 55 LTP (a) and 9 No-LTP (b) channels in response to TBS in 6 DCS-treated slices from 6 WT mice. (c,d) Pooled data for fEPSP slope changes of 43 LTP (c) and 15 No-LTP (d) channels in response to TBS in 6 DCS-treated slices from 5 ASIC1a KO mice. Note, for display purpose, some LTP channels are not shown in (a) due to their extraordinarily high magnitude of acute potentiation or large fluctuation. (e) Time courses of mean fEPSP slope changes in response to TBS of all activated channels in 6 DCS-treated slices from 5-6 mice each for WT (red) and ASIC1a KO (blue) groups. Inset: representative fEPSP traces at the time points indicated by the numbers. Calibration: 100 μV, 10 ms. The mean fEPSP slope values during the baseline period were -45.3 μV/ms for WT and -45.8 μV/ms for KO group. (f) Summary data (number/slice, n = 6 slices/5-6 mice) of activated channels that responded to Schaffer collateral pathway stimulation and LTP channels that responded to TBS in the presence of DCS in the CA1 regions of WT (filled red bar) and ASIC1a KO (filled blue bar) mice. The data from Fig. S3f were re-plotted here for comparison (stripped red and blue bars). ***$P < 0.001$ for ASIC1a KO vs. WT, unpaired Student’s t-test. Arrows in (a-e) indicate the TBS delivery. Horizontal bars in (a-e) denote the application of DCS. Error bars in (e,f) represent SEM.

Supplementary Figure S5

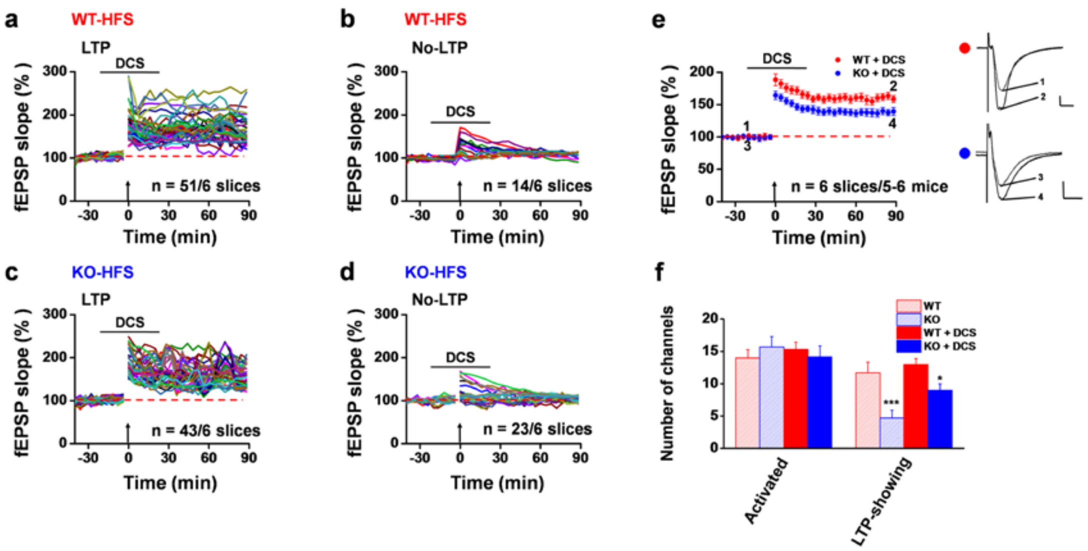

Supplementary Figure S5. A higher dose of DCS still could not fully rescue the impaired HFS-evoked LTP in hippocampal slices from ASIC1a KO mice

(a,b) Pooled data for fEPSP slope changes of 51 LTP (a) and 14 No-LTP (b) channels in response to HFS in 6 slices treated with DCS (100 μM, 40 min) from 6 WT mice. (c,d) Pooled data for fEPSP slope changes of 43 LTP (c) and 23 No-LTP (d) channels in response to HFS in 6 slices treated with DCS from 5 ASIC1a KO mice. (e) Time courses of mean fEPSP slope changes in response to HFS of all activated channels in 6 DCS-treated slices from 5-6 mice each for WT (red) and ASIC1a KO (blue) groups. Inset: representative fEPSP traces at the time points indicated by the numbers. Calibration: upper, 100 μV, 10 ms; lower, 50 μV, 10 ms. The mean fEPSP slope values during the baseline period were -40.0 μV/ms for WT and -35.9 μV/ms for KO group. (f) Summary data (number/slice, n = 6 slices/5-6 mice) of activated channels that responded to Schaffer collateral pathway stimulation and LTP channels that responded to HFS in the presence of DCS in the CA1 regions of WT (filled red bar) and ASIC1a KO (filled blue bar) mice. The data from Fig. 2h were re-plotted here for comparison (stripped red and blue bars). ***$P < 0.001$ for ASIC1a KO vs. WT; *$P < 0.05$ for ASIC1a KO + DCS vs. WT + DCS, unpaired Student's t-test. Arrows in (a-e) indicate the HFS delivery. Horizontal bars in (a-e) denote the application of DCS. Error bars in (e,f) represent SEM.

Supplementary Figure S6

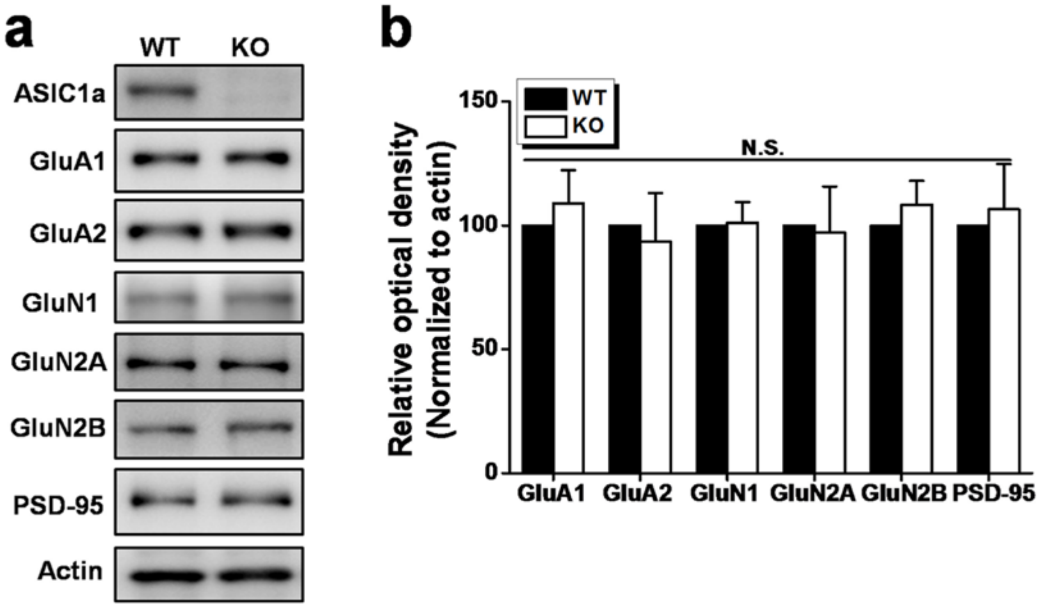

Supplementary Figure S6. Genetic deletion of ASIC1a has no effect on the synaptic expression of glutamate receptor subunits in the hippocampus

Synaptic expression of various glutamate receptor subunits in the hippocampus of WT and ASIC1a null mice was determined by Western blotting of postsynaptic density (PSD) fractions. (a) Representative Western blots for ASIC1a, GluA1, GluA2, GluN1, GluN2A, GluN2B and PSD95 in the PSD fractions of hippocampi obtained from WT and ASIC1a KO mice. (b) Pooled data from several experiments as shown in (a). Data represent mean ± S.E.M. n = 5 for each group. N.S., not significant, WT vs. ASIC1a KO, paired Student's t test. Please note that cropped gels are used in (a) and full-length blots are shown in supplementary Figure S7. All gels were run under the same experimental conditions.

Supplementary Figure S7

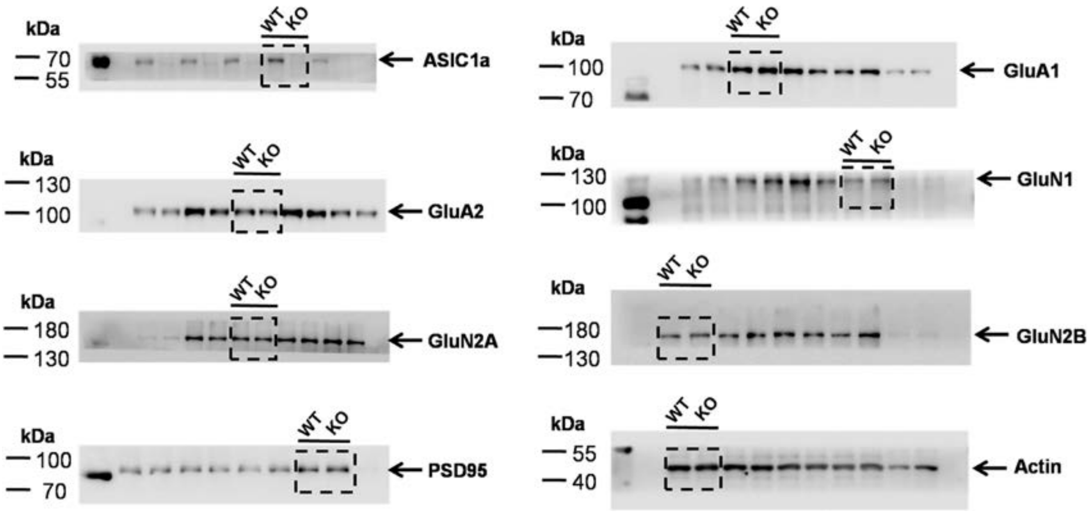

Supplementary Figure S7. Original images of full length blots presented in supplementary Figure S6.
